# Multiomics Analysis of Structural Magnetic Resonance Imaging of the Brain and Cerebrospinal Fluid Metabolomics in Cognitively Normal and Impaired Adults

**DOI:** 10.3389/fnagi.2021.796067

**Published:** 2022-01-25

**Authors:** Ronald C. Eldridge, Karan Uppal, Mahsa Shokouhi, M. Ryan Smith, Xin Hu, Zhaohui S. Qin, Dean P. Jones, Ihab Hajjar

**Affiliations:** ^1^Nell Hodgson Woodruff School of Nursing, Emory University, Atlanta, GA, United States; ^2^Division of Pulmonary, Allergy, Critical Care, and Sleep Medicine, School of Medicine, Emory University, Atlanta, GA, United States; ^3^Department of Neurology, School of Medicine, Emory University, Atlanta, GA, United States; ^4^Department of Biostatistics and Bioinformatics, Rollins School of Public Health, Emory University, Atlanta, GA, United States

**Keywords:** MRI imaging, metabolomics (OMICS), multiomics analysis, mild cognition impairment, amino acids (AA), Alzheimer’s disease, integrative “omics,”, gray matter atrophy

## Abstract

**Introduction:**

Integrating brain imaging with large scale omics data may identify novel mechanisms of mild cognitive impairment (MCI) and early Alzheimer’s disease (AD). We integrated and analyzed brain magnetic resonance imaging (MRI) with cerebrospinal fluid (CSF) metabolomics to elucidate metabolic mechanisms and create a “metabolic map” of the brain in prodromal AD.

**Methods:**

In 145 subjects (85 cognitively normal controls and 60 with MCI), we derived voxel-wise gray matter volume via whole-brain structural MRI and conducted high-resolution untargeted metabolomics on CSF. Using a data-driven approach consisting of partial least squares discriminant analysis, a multiomics network clustering algorithm, and metabolic pathway analysis, we described dysregulated metabolic pathways in CSF mapped to brain regions associated with MCI in our cohort.

**Results:**

The multiomics network algorithm clustered metabolites with contiguous imaging voxels into seven distinct communities corresponding to the following brain regions: hippocampus/parahippocampal gyrus (three distinct clusters), thalamus, posterior thalamus, parietal cortex, and occipital lobe. Metabolic pathway analysis indicated dysregulated metabolic activity in the urea cycle, and many amino acids (arginine, histidine, lysine, glycine, tryptophan, methionine, valine, glutamate, beta-alanine, and purine) was significantly associated with those regions (*P* < 0.05).

**Conclusion:**

By integrating CSF metabolomics data with structural MRI data, we linked specific AD-susceptible brain regions to disrupted metabolic pathways involving nitrogen excretion and amino acid metabolism critical for cognitive function. Our findings and analytical approach may extend drug and biomarker research toward more multiomics approaches.

## Introduction

Advancements in brain imaging and omics fields (genomics, transcriptomics, proteomics, etc.) have produced a wealth of knowledge regarding the underlying biology of cognitive decline in the healthy aging population and those who suffer from neurocognitive disorders such as Alzheimer’s disease (AD), Parkinson’s, and dementia (Johnson et al., 2012; Sancesario and Bernardini, 2018). Merging imaging and omics modalities may provide further breakthroughs not attainable by a single field to aid patient prognosis, precision medicine, early detection and prevention of neurocognitive decline (Avramouli and Vlamos, 2017; Pimplikar, 2017; Antonelli et al., 2019).

Brain imaging is one way to assess the structural changes preceding and during neurocognitive decline. Brain atrophy (i.e., the loss of gray matter), as measured by magnetic resonance imaging (MRI), can reflect a patient’s progression from normal cognition to mild cognitive impairment (MCI) and AD (Bozzali et al., 2006; Matsuda, 2016). Whole-brain voxel-wise measurements of gray matter volume is now common. The approach provides a comprehensive and less biased assessment of anatomical and volumetric differences between cognitively impaired individuals and cognitively normal controls (Ries et al., 2008; Matsuda, 2013).

High-resolution untargeted metabolomics (HRM) is the newest entrant to the omics fields. It uses liquid or gas-chromotography paired with high-throughput mass-spectrometry to comprehensively measure endogenous small biochemical compounds and signaling molecules (i.e., metabolites) (Liu and Locasale, 2017) as well as exogenous environmental chemical exposures (i.e., the exposome) (Vermeulen et al., 2020). Given that metabolic dysfunction in the brain and surrounding tissues plays a significant role in the onset and progression of cognitive impairment (de la Monte and Tong, 2014; Campos-Peña et al., 2017; Szablewski, 2017), untargeted metabolomics is primed to make key discoveries about the pathological mechanisms underlying MCI and AD (Wilkins and Trushina, 2018).

For this project, we integrated voxel-wise MRI brain imaging with untargeted metabolomics in cerebrospinal fluid (CSF) to study the shared metabolic and structural mechanisms of mild cognitive impairment (MCI) – i.e., to create a CSF metabolic map of the MCI vs. cognitively normal brain. Each method has made individual discoveries regarding the biology of cognitive decline, however, to our knowledge, this is the first study to perform large-scale integration of the two. Since this is novel, there is no established workflow. Our analytical approach is just one of many possible approaches. Nevertheless, a multiomics analysis of the two data sources may indeed provide useful information not attainable by either method separately.

## Materials and Methods

### Participant Cohort

The cohort is part of a larger NIH consortium, Molecular Mechanisms of the Vascular Etiology of Alzheimer’s Disease (M2OVE-AD). More information about M2OVE-AD can be found here: https://adknowledgeportal.synapse.org/. Specific information on the data used for this analysis can be found here: https://adknowledgeportal.synapse.org/Explore/Studies/DetailsPage?Study=syn18909507. Subjects were recruited via the Brain Stress Hypertension and Aging Program (B-SHARP) at Emory University. B-SHARP participants underwent baseline cognitive assessments by a study physician, neuroimaging and lumbar punctures before being enrolled in observational studies or clinical trials based on their eligibility and consent. Participants were excluded if they had a history of stroke in the past 3 years, were unwilling or unable to undergo study procedures including MRI and LP, did not have a study informant, had a clinical diagnosis of dementia of any type, or abnormal serum Thyroid Stimulating Hormone (>10) or B12 (<250). Recruitment occurred through referral from the Goizueta Alzheimer’s Disease Research Center at Emory or through community partnerships with health education organizations, health fairs, advertisements and mailed announcements. The current analysis used data from 145 subjects (60 with MCI and 85 cognitively normal controls) enrolled in B-SHARP from March 2016-January 2019 who had baseline data, whole brain MRI imaging, and CSF metabolomics.

### Cognitive Diagnosis

Mild cognitive impairment (MCI) categorization was done using a modified Peterson criteria with the Montreal Cognitive Assessment (MoCA) instead of Mini-Mental State Exam. MCI criteria included subjective memory complaints, a MoCA < 26, Clinical Dementia Rating (CDR) score, memory sum of boxes = 0.5, education adjusted cutoff score on Logical Memory delayed recall of the Wechsler Memory Scale and preserved Functional Assessment Questionnaire (FAQ) ≤ 7. Normal cognition was defined as having no significant memory complaints beyond those expected for age, a MoCA score > 26 points, a CDR score of 0 (including a 0 on the Memory Box score), and preserved FAQ ≤ 7. Each subject underwent a review with the study physician and PI (Hajjar) and a neuropsychologist. When the participant’s assessment did not reveal a clear cognitive category, a consensus diagnosis was sought by the study PI and the neuropsychologist reviewing the physician interview, cognitive assessment, and other relevant elements. If the two evaluators failed to reach an agreement, then a third independent cognitive neurologist from Emory, blinded from the initial diagnosis, was consulted and his assessment broke the tie.

### Magnetic Resonance Imaging Acquisition and Processing

Magnetic resonance imaging was performed using 3.0 Tesla scanner (Magnetom Prisma, Siemens, Erlangen, Germany). Anatomical T1-weighted images were acquired using high-resolution three-dimensional magnetization-prepared rapid acquisition with gradient echo (MPRAGE). Images were then digitally saved for offline processing. For each scan, preprocessing was performed using voxel-based morphometry toolbox (Ashburner, 2007) in the SPM package and the results were visually inspected. Briefly, T1 images were corrected for bias field, and segmented into three separate probability maps: gray matter, white matter, and cerebrospinal fluid. The segmented maps of all subjects were normalized to the MNI template and smoothed using an 8 mm Gaussian filter. We used the voxel-based gray matter volume in our statistical analysis. To this end, the gray matter probability map threshold was 0.3 to remove voxels with less than 30% probability of belonging to gray matter, thus producing a binary gray matter mask. The three-dimensional gray matter mask was transformed to a one-dimensional array for statistical analyses. An inverse transformation was used to convert the statistical results back to the original image space.

### High-Resolution Untargeted Metabolomics

Our HRM approach used an established liquid-chromatography mass spectrometry (LC-MS) workflow developed at the Emory Clinical Biomarkers Laboratory (Accardi et al., 2016; Chandler et al., 2016). CSF aliquots were removed from storage at −80°C and thawed on ice. A 65 μL aliquot of CSF was treated with 130 μL of LC-MS grade acetonitrile, equilibrated for 30 min on ice and centrifuged (16.1 × *g* at 4°C) for 10 min to remove precipitated proteins. The supernatant was added to an autosampler vial and maintained at 4°C until analysis. Sample extracts were analyzed using liquid chromatography and Fourier transform high-resolution mass spectrometry (Dionex Ultimate 3000, Q-Exactive HF, Thermo Scientific). For each sample, triplicate 10 μL injections were analyzed using hydrophilic interaction liquid chromatography (HILIC) with electrospray ionization (ESI) source operated in positive mode. Analyte separation was accomplished using a 2.1 mm × 100 mm × 2.6 μm Accucore HILIC column (Thermo Scientific) and an eluent gradient (A = 2% formic acid, B = water, C = acetonitrile) consisting of an initial 1.5 min period of 10% A, 10% B, 80% C, followed by linear increase to 10% A, 80% B, 10% C at 6 min and then held for an additional 4 min, resulting in a total runtime of 10 min per injection. Mobile phase flow rate was held at 0.35 mL/min for the first 1.5 min, increased to 0.5 mL/min and held for the final 4 min.

The high-resolution mass spectrometer was operated in full scan mode at 120,000 resolution and mass-to-charge ratio (*m/z*) range 85.0000–1275.0000. Probe temperature, capillary temperature, sweep gas and S-Lens RF levels were maintained at 200°C, 300°C, 1 arbitrary units (AU), and 45 AU, respectively, for both polarities. Positive tune settings for sheath gas, auxiliary gas, sweep gas and spray voltage setting were 45 AU, 25 AU and 3.5 kV, respectively. Raw data was extracted and aligned using apLCMS (Yu et al., 2013) and xMSanalyzer (Uppal et al., 2013) (see Supplementary Laboratory Methods). Uniquely detected ions consisted of accurate mass *m/z*, retention time and ion abundance, referred to as *m/z* features. Data filtering was performed to remove *m/z* features with median coefficient of variation (CV) within technical replicates ≥ 75%. Additionally, only samples with Pearson correlation within technical replicates ≥ 0.7 were used for downstream analysis. Feature intensities for triplicates were median summarized with the requirement that at least two replicates had non-missing values. Batch-effect correction was performed using ComBat (Johnson et al., 2007). Metabolites with <20% missing in either the MCI or cognitively normal populations were imputed with half of the lowest recorded intensity value; metabolites with >20% missing were excluded. Metabolite intensities were log_2_-transformed, and quantile normalized (see Supplementary Figure 1). Specific metabolites pertaining to our findings were annotated by matching mz and retention time to previous confirmed metabolites via laboratory reference standards (Liu et al., 2020) or by a computational match using xmsAnnotator (Uppal et al., 2017). xmsAnnotator is a multistage clustering algorithm that uses metabolic pathway associations, intensity profiles, retention time, mass defect, and adduct patterns to match mz features to publicly available metabolic databases.

### Statistical and Bioinformatic Analysis

We summarize our analytical pipeline in three steps: (1) We performed feature selection by identifying the subset of brain imaging voxels in which gray matter atrophy was associated with MCI vs. controls independent of the CSF metabolomics data. This was done to target the metabolomics integration in step 2 to the areas of the brain most affected by cognitive decline. (2) We then conducted a multiomics analysis of the Step 1 selected voxels with the metabolomics data, thus, finding a set of CSF metabolites that correlated with specific brain regions associated with MCI. (3) We conducted metabolic pathway enrichment analysis of the metabolites found for each cluster in Step 2 to determine dysregulated metabolic pathways specific to each MCI-associated brain region. Lastly, given the large number of voxel-metabolite links we estimated in Step 2, we conducted a sensitivity analysis using training (67%) and validation (33%) sample subsets to investigate the degree to which the multiomics integration findings are replicable (i.e., how robust are the Step 2 findings).

For Step 1, we used threefold cross-validated partial least squares discriminant analysis (PLS-DA) to identify the subset of brain voxels with less gray matter in MCI vs. control subjects (xmsPANDA R program version 3.5.1 using default parameter selections). PLS-DA is a popular multivariate data dimension reduction and feature selection algorithm to distinguish between two or more outcome classes (e.g., MCI vs. control) using linear combinations of the explanatory variables (e.g., voxels), similar to linear discriminant analysis or principal component analysis (Brereton and Lloyd, 2014). PLS-DA is used extensively in metabolomics (Gromski et al., 2015) and is gaining popularity as an analytical tool discriminating cognitive decline with functional and structural MRI data (Andersen et al., 2012; Khedher et al., 2015). Feature selection via PLS-DA was done by ranking the explanatory variables according to their importance at separating the outcomes, called the Variable Importance in Projection (VIP); unfortunately, PLS-DA does not produce *P*-values, nor does it allow for traditional covariate adjustment (e.g., age and sex adjusted estimates). We explored the potential impact of confounding by age and sex by investigating the association of those factors with the CSF metabolites among the cognitively normal controls without incorporating the MRI imaging data. For consideration of multiple comparisons, we pre-specified a VIP cutoff ≥ 2 (a common but strict threshold used in clinical omics research) (Pérez-Enciso and Tenenhaus, 2003) and added the additional restriction of >2% mean difference in gray matter volume. The voxels that met these criteria but had higher gray matter in MCI subjects vs. controls were considered data artifacts and were excluded from the additional analyses for three reasons: (i) lack of prior evidence suggesting MCI is associated with higher gray matter, (ii) the limited number (*n* = 49), and (iii) the fact they did not map to a contiguous and discernible area of the brain. For the voxels that had lower gray matter volume present in MCI subjects vs. controls (*n* = 2,375), we mapped them back to the MNI brain template to identify specific brain regions and subsequently used them for the multiomics analysis.

For Step 2, we integrated voxel-level data from the brain imaging voxels from Step 1 with the extracted CSF metabolomics data using a publicly available multiomics network algorithm, xMWAS (Uppal et al., 2018). xMWAS is an R program that automates existing network algorithms to identify and graph clusters of correlated data from multiple sources (e.g., clusters of imaging voxels and metabolites). It uses sparse partial least squares regression, community detection algorithms and eigen vector centrality measures to estimate pair-wise correlations between voxels and metabolites and evaluate unique voxel-metabolite clusters. The program identifies the number of voxel-metabolite clusters by optimizing cluster modularity, a frequently used community detection algorithm that partitions a network into clusters made up of densely connected nodes (i.e., voxels and metabolites), so that nodes belonging to different clusters are sparsely connected (Blondel et al., 2008). xMWAS requires the researcher to set a Pearson correlation cutoff to model network links. We set ours at >|0.271| with a *P*-value < 0.05 because that was the weakest correlation in which each of the 2,375 imaging voxels correlated with ≥1 metabolite. We then annotated the specific brain regions that corresponded to each imaging-metabolite cluster.

For Step 3, we performed metabolic pathway enrichment analysis on the metabolites in each imaging-metabolite cluster to describe dysregulation of metabolic pathways specific for each brain region identified in Step 2. This was done using the default inputs (i.e., Human model, Mass accuracy = 10 ppm, Adducts: M[1 + ], M + H[1 + ], and M + Na[1 + ]) for the online version of Mummichog 2.0, an untargeted metabolomics pathway analysis tool (Li et al., 2013). Mummichog requires two metabolite lists: a user-specified list of significant metabolites (e.g., the metabolites in imaging-metabolite cluster 1) and a list of non-significant reference metabolites (e.g., the whole list of extracted metabolites). Mummichog constructs the metabolic pathways by mapping the reference list to the Kyoto Encyclopedia of Genes and Genomes (KEGG) database and searches for enrichment from the user-specified list. Mummichog calculates a Fisher’s exact P-value for each metabolic pathway via permutation testing using repeated random sampling from the referenced list. Each imaging-metabolite cluster was investigated separately for pathway enrichment to describe dysregulated metabolic pathways specific for each brain region found in Step 2.

For the sensitivity analysis, we randomly divided our full sample size into a 67% training set (*n*_cases_ = 40, *n*_controls_ = 57) and a 33% validation set (*n*_cases_ = 20, *n*_controls_ = 28). Among the training set, we re-ran the Step 2, voxel-metabolite integration using the same xMWAS parameters as we used in the full dataset (*r* > |0.271|with a *P*-value < 0.05). We compared the xMWAS voxel-metabolite pair-wise correlations found in the training set against identical xMWAS voxel-metabolite correlations in the validation set; we also investigated the individual and average voxel-metabolite correlations of just the 20 metabolites we found in the Step 3 pathway analysis. For each investigation, we provide a scatter plot with a fitted linear regression and *R*^2^ value to demonstrate the degree to which the xMWAS findings in the training set were replicated in the validation set.

## Results

Of the 145 subjects, 60 had MCI and 85 controls were cognitively normal (Table 1). Subjects with MCI were older (67.1 average years vs. 62.7) and were more likely to be male (42% vs. 29%). As expected, subjects with MCI had lower cognitive performance and less total hippocampal volume than the controls. Though the average age significantly differed between MCI vs. controls, age was generally associated with different metabolites and different metabolic pathways than we found in our primary results in Step 2 and 3 (see Confounding by Age and Sex in the Supplemental). Based on this we concluded that age was not a confounder of our primary results. Sex also differed between MCI vs. controls but was generally associated with different metabolites than our primary results. It was, however, weakly associated with two amino acid pathways we found in our primary results: histidine metabolism and methionine and cysteine metabolism. Based on these results we concluded that sex was a weak confounder that does not meaningfully change our primary results or conclusions.

**TABLE 1:** Characteristics of the sample stratified by cognitive diagnosis: normal cognition and mild cognitive impairment (MCI).

		Normal cognition	MCI	
Characteristic		(*n* = 85)	(*n* = 60)	*p*-value
Age in years (±SD)		62.7 ± 7.1	67.1 ± 9.2	0.001
Sex				0.13
	Female	60(70.6%)	35(58.3%)	
	Male	25(29.4%)	25(41.7%)	
Race				0.23
	Non-Hispanic White	59(69.4%)	35(58.3%)	
	African American	25(29.4%)	25(41.7%)	
	Other	1(1.2%)	0(0.0%)	
Education in years (±SD)		16.5 ± 2.6	16.0 ± 2.8	0.27
BMI				0.24
	Underweight	0(0.0%)	3(5.0%)	
	Normal	36(42.9%)	23(38.3%)	
	Overweight	30(35.7%)	19(31.7%)	
	Obese	15(17.9%)	14(23.3%)	
	Morbid Obesity	3(3.6%)	1(1.7%)	
CDR				<0.001
	0	83(98.8%)	7(11.7%)	
	0.5	1(1.2%)	53(88.3%)	
MoCA				<0.001
	≥26	64(75.3%)	1(1.7%)	
	<26	21(24.7%)	59(98.3%)	
Family history of AD				0.35
	No	34(40.5%)	29(48.3%)	
	Yes	50(59.5%)	31(51.7%)	
Hypertension				0.006
	No	36(43.4%)	40(66.7%)	
	Yes	47(56.6%)	20(33.3%)	
Diabetes				0.78
	No	72(86.7%)	53(88.3%)	
	Yes	11(13.3%)	7(11.7%)	
Heart disease				0.63
	No	68(80.0%)	46(76.7%)	
	Yes	17(20.0%)	14(23.3%)	
High cholesterol				0.44
	No	43(51.8%)	35(58.3%)	
	Yes	40(48.2%)	25(41.7%)	
Stroke				0.96
	No	79(95.2%)	57(95.0%)	
	Yes	4(4.8%)	3(5.0%)	
Total lesion volume in mm^3^ (±SD)		1.5 ± 2.1	3.3 ± 4.9	0.02
Total hippocampal volume in mm^3^ (±SD)		7641 ± 876	6791 ± 1077	<0.001

*SD, standard deviation; BMI, body mass index; AD, Alzheimer’s disease; CDR, clinical dementia rating; MoCA, Montreal cognitive assessment.*

The whole-brain MRI produced 247,941 unique voxels for each subject. An unbiased voxel-wise approach using PLS-DA identified 2,424 voxels (approximately 1%) that – having met the pre-specified criteria of VIP ≥ 2 and > 2% mean difference in gray matter – differentiated between MCI and controls in our population (PLS-DA score plots are in Supplementary Figure 2 and a Volcano plot is in Supplementary Figure 3). Of those, 49 voxels indicated higher gray matter in MCI subjects and were excluded from further analyses. The remaining 2,375 voxels indicated lower gray matter in MCI subjects and were used for the multiomics analysis. The 2,375 voxels included contiguous clusters in regions known to be susceptible in the neuropathology of MCI and AD: the hippocampus, parahippocampal gyrus, thalamus, orbitofrontal cortex and visual cortex (Supplementary Figure 4).

**FIGURE 1:**
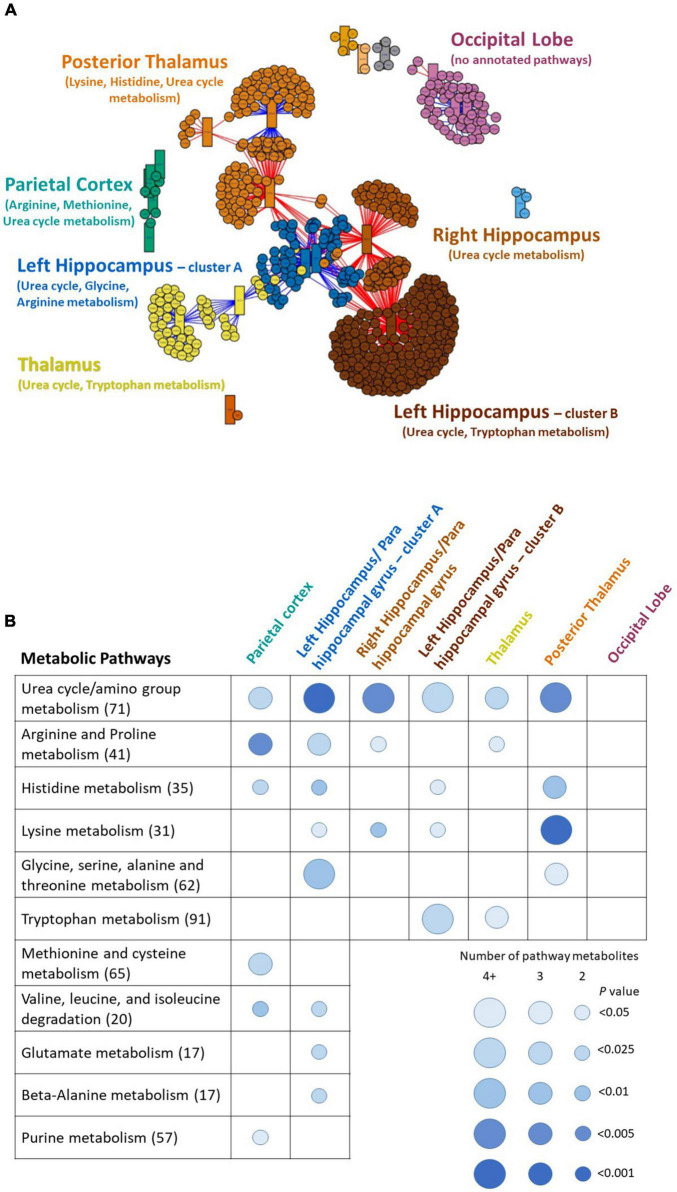
**(A)** Multiomics clustering network of MRI-derived gray matter brain imaging voxels and CSF metabolites. The network algorithm (xMWAS) identified twelve distinct imaging-metabolite clusters. Each cluster is indicated by a different color. The seven clusters that correspond to contiguous regions of the brain are labeled. The circles are MRI brain imaging voxels and the rectangles are cerebrospinal fluid metabolites. Blue lines indicate positive correlation with controls, red lines indicate negative correlation. This is a modified figure limiting the number of voxels, metabolites, and connections to visualize the clusters and their relationships. The unaltered figure is in Supplementary Figure 5. **(B)** The associated metabolic pathways from the seven annotated imaging-metabolite clusters identified by the multiomics network. Metabolic pathway enrichment analysis (Mummichog) described the dysregulated metabolic pathways specific to each region of the brain. The size and color of the circles indicate the number of significant overlapping metabolic features in the pathway and the Fisher’s exact *P*-value of each pathway. The number next to the metabolic pathway is the number of reference metabolites in each pathway. The metabolites that correlated with the occipital lobe were sparse and did not collectively map to a specific known pathway.

**FIGURE 2:**
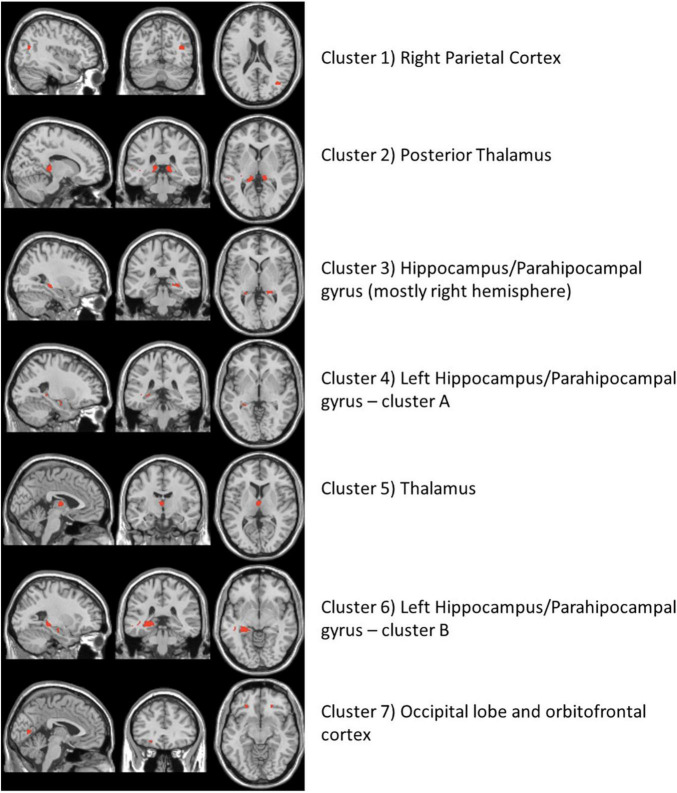
The neuroimaging atlas of the seven annotated brain regions as identified by the imaging metabolite clusters from the multiomics network algorithm, xMWAS. The red coloring shows the location of imaging voxels for each cluster.

The high-resolution untargeted metabolomics extracted 13,064 unique CSF metabolic features according to mass to charge (m/z) and retention time. After filtering for missing data, 9,804 features remained for all 145 subjects and were used for analysis. The integration of the brain imaging data with the CSF metabolomics data generated twelve unique imaging-metabolite clusters by correlating all 2,375 imaging voxels with 463 of the 9,804 metabolic features (Table 2, Figure 1A, and Supplementary Table 3); 9,341 metabolites did not correlate with an imaging voxel. Seven of those clusters corresponded to contiguous brain regions important to neurocognitive decline: (1) Parietal cortex; (2) Posterior Thalamus; (3) Hippocampus/Parahippocampal gyrus (mostly right hemisphere); (4) Left Hippocampus/Parahippocampal gyrus (cluster A); (5) Thalamus; (6) Left Hippocampus/Parahippocampal gyrus (cluster B); (7) Occipital lobe and orbitofrontal cortex (bilateral) (Figure 2 and Supplementary Figure 4). The remaining five clusters were small (average voxel size = 12.8) and did not conform to contiguous regions of brain in the MNI image and were not further analyzed. The five clusters corresponding to the hippocampus and thalamus shared connections with some of the same metabolites (i.e., hippocampal voxel–metabolite–thalamus voxel correlations). Conversely, the clusters that corresponded to the parietal cortex and the occipital lobe were separate.

**TABLE 2:** The number of voxels and metabolites that comprise each community cluster identified by the xMWAS multiomics analysis program when integrating 2,375 voxels with 463 metabolites.

	Brain region	# of Voxels	# of Metabolites
Cluster 1	Right parietal cortex	90	111
Cluster 2	Posterior thalamus	406	96
Cluster 3	N/A^a^	11	13
Cluster 4	Hippocampus/ Parahippocampal gyrus (right hemisphere)	209	68
Cluster 5	N/A^a^	7	14
Cluster 6	Left Hippocampus/ Parahippocampal gyrus – cluster A	438	108
Cluster 7	Thalamus	158	54
Cluster 8	Left Hippocampus/ Parahippocampal gyrus – cluster B	617	107
Cluster 9	N/A^a^	13	36
Cluster 10	Occipital lobe and orbitofrontal cortex	393	177
Cluster 11	N/A^a^	32	31
Cluster 12	N/A^a^	1	1
**Total**		2,375	816^b^

*^a^Not applicable because the voxels did not conform to a contiguous region of the brain in the MNI image.*

*^b^The total is larger than 463 because many metabolites linked to >1 cluster.*

We conducted metabolic pathway enrichment analyses among the CSF metabolites that correlated with each of the seven brain regions that differentiated MCI vs. controls. We summarize the statistically significant dysregulated pathways and their linked brain regions in Figure 1B; the complete list is in Supplementary Tables 4–10. In six of the regions (hippocampus [3 regions], thalamus [2], and parietal cortex) we found significant dysregulation of CSF metabolic activity that regulates the urea cycle, and a host of amino acids (arginine, histidine, lysine, glycine, tryptophan, methionine, valine, glutamate, beta-alanine, and purine). Dysregulation of urea cycle metabolism was the most significant and consistent finding across the six regions. Notably, the metabolites that correlated with the occipital lobe did not generate an enriched metabolic pathway according to Mummichog. This may be due to an inherent limitation of current knowledge of metabolic pathways. In Table 2, we list the 20 metabolites that comprised the statistically significant metabolic pathways linked to the brain regions. We include the metabolite annotation and note those that matched to lab-confirmed reference standards (Uppal et al., 2017; Liu et al., 2020), the confidence level in that identification per Schymanski et al. (2014) the MCI vs. cognitively normal fold change, and correlation links to MRI voxels and brain regions. Among those, some notable metabolites and their relative levels in MCI subjects vs. controls are amino butyrate* (lower in MCI), histamine* (lower), creatinine (higher), guanidinoacetate* (lower), hypoxanthine (higher), 5-hydroxyindoleacetate* (higher); *indicates a laboratory confirmed metabolite.

**FIGURE 3:**
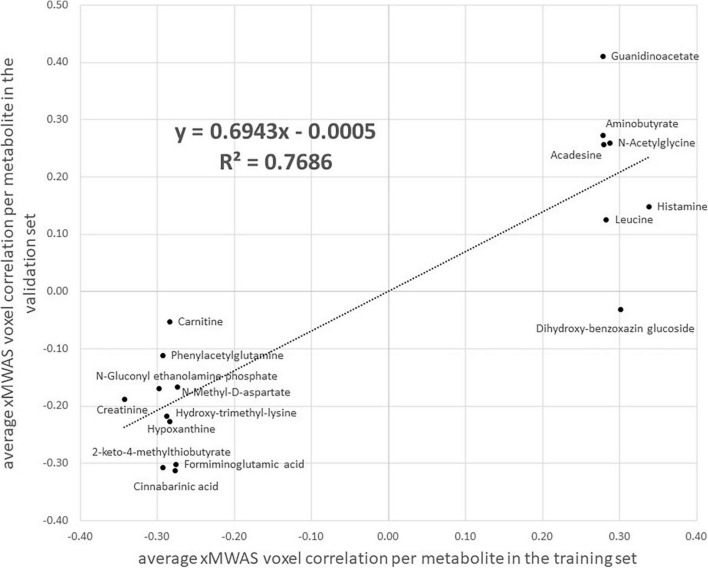
A scatter plot of the average correlation, as estimated by xMWAS, between MRI imaging voxels and 17 amino acid/urea cycle metabolites found via metabolic pathway analysis that met the xMWAS parameter thresholds (*r* > | 0.271| with a *P*-value < 0.05) in the training set. The *x*-axis is the average correlation in the training set and the *y*-axis is the average correlation in the validation set. Three metabolites, indoleacetic acid, 5-hydroxyindoleacetate, and *S*-adenosylhomocysteine from Table 3 did not meet the xMWAS parameter thresholds in the training set and were thus not included.

Using the same input data and parameter selections, the Step 2 multiomics integration sensitivity analysis integrated all 2,375 voxels with 1,638 metabolites among the randomly selected training set. Comparing the xMWAS voxel-metabolite correlations found in the training set with identical ones in the validation set, we found low correlation (*r* = 0.34, *R*^2^ = 0.11; Supplementary Figure 6), suggesting that many of voxel-metabolite links might not be replicable. However, when we restricted the comparison to the voxel-metabolite links of the metabolites we found in the Step 3 pathway analysis (Table 3), we found a much stronger correlation (*r* = 0.74, *R*^2^ = 0.55; Supplementary Figure 6), suggesting the links xMWAS found between these amino acid/urea cycle metabolites and their corresponding brain regions are more robust. Since many of those amino acid/urea cycle metabolites had multiple voxel connections, we compared the average voxel correlation in the training set with the average voxel correlation in the validation set for each metabolite via a scatter plot (Figure 3). The averages show even stronger correlations between the training set and the validation set (*r* = 0.88, *R*^2^ = 0.77). It should be noted that three of the twenty metabolites, indoleacetic acid, 5-hydroxyindoleacetate, and *S*-adenosylhomocysteine, did not meet the xMWAS parameter criteria (*r* > | 0.271| with a *P*-value < 0.05) to link to voxels in the training set and thus were not compared to the validation set.

**TABLE 3:** Twenty cerebrospinal fluid metabolites that comprise the significant pathways per the Mummichog metabolic pathway analysis.

m/z	Time*^a^*	Metabolite	Associated metabolic pathways	Adduct	ID level*^b^*	Fold change*^c^*	Voxel links*^d^*	Associated areas of the brain
104.0706	277	Gamma-aminobutyrate	Urea cycle/amino group metabolism; Glycine, serine, alanine, and threonine metabolism	M + H	1	–0.18	16	Posterior Thalamus, Hippocampus – left hemisphere (cluster A)
111.0805	157	Histamine	Histidine metabolism	M[1+]	1	-0.28	757	Posterior Thalamus, Hippocampus - left hemisphere (cluster A and B)
115.0632	34	Creatinine	Urea cycle/amino group metabolism; Arginine and Proline Metabolism	M + H[+1]	3	0.13	1,374	Parietal Cortex, Posterior Thalamus, Hippocampus – right and left hemisphere (cluster A and B), Thalamus
118.0499	160	*N*-acetylglycine	Urea cycle/amino group metabolism; Glycine, serine, alanine and threonine metabolism	M + H	1	–0.10	8	Hippocampus – left hemisphere (cluster A and B)
118.0604	205	Guanidinoacetate (Glycocyamine)	Urea cycle/amino group metabolism; Arginine and Proline Metabolism; Glycine, serine, alanine and threonine metabolism	M + H	1	–0.22	258	Posterior Thalamus, Hippocampus – right hemisphere, Occipital lobe/Orbitofrontal cortex
132.1019	42	Leucine	Valine, leucine and isoleucine degradation	M + H	1	-0.11	6	Parietal Cortex
137.0458	43	Hypoxanthine	Purine metabolism	M + H	1	0.04	476	Parietal Cortex
148.0605	55	*N*-methyl-D-aspartate	Urea cycle/amino group metabolism; Glycine, serine, alanine, and threonine metabolism; Histidine metabolism; Arginine and Proline Metabolism; Glutamate metabolism; Beta-Alanine metabolism; Valine, leucine and isoleucine degradation; Butanoate metabolism; Lysine metabolism; Aspartate and asparagine metabolism; Methionine and cysteine metabolism; Purine metabolism	M + H	1	-0.03	9	Parietal Cortex, Hippocampus - left hemisphere (cluster A)
149.0281	77	2-keto-4-methylthiobutyrate	Methionine and cysteine metabolism	M + H	4	0.22	66	Parietal Cortex
175.0713	50	Formiminoglutamic acid	Histidine metabolism	M + H	3	0.29	176	Parietal Cortex, Posterior Thalamus, Hippocampus - right and left hemisphere (cluster A and B)
176.0705	189	Indoleacetic acid	Tryptophan metabolism	M + H	4	–0.10	58	Hippocampus – left hemisphere (cluster B)
185.1035	46	Carnitine	Lysine metabolism	M + Na[1+]	5	0.41	1	Posterior Thalamus
192.0655	36	5-Hydroxyindoleacetate	Urea cycle/amino group metabolism, Arginine and Proline metabolism, Tryptophan metabolism	M + H	1	0.08	360	Posterior Thalamus, Hippocampus - right and left hemisphere (cluster A and B), Thalamus
205.1547	283	Hydroxy-trimethyl-lysine	Lysine metabolism	M[1+]	5	0.32	314	Posterior Thalamus, Hippocampus – right and left hemisphere (cluster A and B)
259.1036	45	Acadesine	Tryptophan metabolism	M[1+]	5	0.03	55	Thalamus, Hippocampus – left hemisphere (cluster B)
265.1202	55	Phenylacetylglutamine	Tryptophan metabolism	M + H	4	0.01	9	Thalamus
320.0764	50	*N*-gluconyl ethanolamine phosphate	Urea cycle/amino group metabolism, Arginine and Proline Metabolism; Methionine and cysteine metabolism;	M + H	3	–0.02	608	Parietal Cortex, Hippocampus – right and left hemisphere (cluster A and B), Thalamus
323.0288	97	Cinnabarinic acid	Tryptophan metabolism	M + Na[1 + ]	5	0.01	1	Hippocampus - left hemisphere (cluster B)
344.0957	70	Dihydroxy-benzoxazin glucoside	Lysine metabolism	M + H	4	–0.45	214	Posterior Thalamus, Hippocampus – right and left hemisphere (cluster B)
385.1303	73	*S*-adenosylhomocysteine	Lysine metabolism; Urea cycle/amino group metabolism; Histidine metabolism; Glycine, serine, alanine, and threonine metabolism	M + H	1	0.27	1	Posterior Thalamus

*^a^Metabolite extraction retention time in seconds.*

*^b^The level of confidence in each metabolite annotation per Schymanski et al. (2014): 1, lab confirmed structure by reference standards; 3, tentative candidate via a high confident computational match to human metabolome database; 4, unequivocal molecular formula via a medium confidence computational match; 5, exact mass of interest via a low confidence computational match; computational matches made by xmsAnnotator (Uppal et al., 2017).*

*^c^Log_2_ fold change – positive numbers mean higher levels found in MCI cases vs. controls, negative numbers mean lower levels found MCI cases vs. controls.*

*^d^The number of network links with imaging voxels at Pearson’s r ≥ |0.271|.*

## Discussion

By integrating whole-brain structural MRI with CSF untargeted metabolomics, we created a novel metabolic map to MCI-relevant brain regions. We found lower levels of gray matter in the hippocampus, thalamus, and parietal cortex to be associated with dysregulated urea cycle and amino acid metabolic pathways in CSF. Since gray matter atrophy in those regions is an early marker for MCI and AD our study indicates that dysregulated nitrogen excretion and amino acid metabolism may play an important role in early neurocognitive decline. While MRI imaging and, to a lesser extent, metabolomics have been previously studied in MCI and AD, a multiomics analysis of these large data sources has provided novel insight to help guide future biomarker and therapeutic research.

The hippocampus and surrounding regions are vulnerable to neurodegeneration in the pathology of cognitive decline (Ries et al., 2008; Tabatabaei-Jafari et al., 2015; Schröder and Pantel, 2016; Yi et al., 2016). Prior studies have shown gray matter atrophy in the hippocampus, as measured by voxel-based MRI, to be a discriminating biomarker of the progression from normal cognition to MCI to AD (Klöppel et al., 2008; Cuingnet et al., 2011; Retico et al., 2015). Our whole-brain voxel-wise analysis reinforces the importance of gray matter loss in the hippocampal region as a marker of MCI but suggests additional loss in the thalamus, orbitofrontal cortex and visual cortex may also be relevant.

We believe our study is the first to comprehensively integrate and analyze untargeted CSF metabolomics with structural MRI brain imaging data, thus complicating the task of comparing our results to prior studies. Further complicating the matter is that untargeted metabolomics is methodologically and analytically different from targeted metabolomics, which is a more common method (Trushina and Mielke, 2014). In a recent untargeted metabolomics study of CSF in MCI cases and cognitively normal controls that did not use MRI imaging, Trushina et al. (2013) reported dysregulation of metabolic pathways that we did not observe, such as the TCA cycle, steroid hormones, and neurotransmitters, but also the urea cycle and many amino acids that we did observed. Where our findings differ from Trushina et al. (2013) may demonstrate the novelty and utility of our multiomics approach. Dysregulated urea cycle and amino acid metabolism may be biologically relevant to hippocampal and thalamus gray matter neurodegeneration, while dysregulated metabolism in other pathways (e.g., sugars, hormones, nucleotides, etc.) may relate to other aspects of neurocognitive cognitive decline.

Seiler (2002) suggested ammonia as a contributing factor to the pathogenesis and progression of AD. Ammonia (NH_3_) is a nitrogen-based compound that is created by the metabolism of amino acids. Because it is neurotoxic (Enns, 2008), excess nitrogen and ammonia in brain tissue are reconstituted and transported to the liver to be excreted via the urea cycle. Seiler theorized that high levels of ammonia to be a cause of neurodegeneration since he noted that ammonia metabolism is impaired in AD patients, and consequently ammonia levels are elevated. The findings in our study support Seiler’s hypothesis. Targeted metabolomics studies have also found an association between urea cycle and amino acid metabolites and AD (González-Domínguez et al., 2015; Kori et al., 2016; Chouraki et al., 2017). One theory is that increased dependence on cerebral amino acid metabolism is the result from impaired glucose energy metabolism, an early precursor to AD (Griffin and Bradshaw, 2017). In otherwise healthy individuals, the excess ammonia and nitrogen stemming from amino acid metabolism may be managed. However, in MCI and prodromal AD patients, urea cycle and amino acid metabolic dysregulation may accelerate a cascade of neuronal changes that further the progression of AD.

A primary goal of a large-data multiomics analysis such as ours, is to gain insight not attainable by either dataset alone. For example, a prior CSF metabolomics study conducted by our research group that had an overlapping study population but did not use MRI brain imaging showed slightly different results. In that study, we found sugar metabolism (i.e., *N*-glycan, sialic acid, aminosugars, and galactose) as the primary dysregulated metabolic pathway distinguishing MCI from cognitively normal subjects; the urea cycle and some amino acid pathways were statistically significantly dysregulated but weaker in magnitude (Hajjar et al., 2020). Though abnormal cerebral glucose metabolism is critically important to AD pathogenesis (Chen and Zhong, 2013), the integration of structural MRI data in this analysis reveals the importance of the urea cycle and amino acid metabolism specifically to neurodegeneration in the hippocampus and thalamus.

Key limitations to this study include the cross-sectional design, the incomplete identification of metabolic features and pathways common to untargeted metabolomics analyses, and the approach which lacked covariate adjustment due to the multivariate integration programs. The cross-sectional design precludes a temporal relation thus we do not know whether urea cycle and amino acid metabolism may be a potential cause or just a biomarker of neurodegeneration. Longitudinal studies will be needed to tease apart this issue. Moreover, the difficulty of metabolite identification using an untargeted approach is a current limitation of the field (Uppal et al., 2016). To compensate, we reported the confidence level for our annotated metabolites using confirmed authentic standards (Liu et al., 2020) and state-of-the-art computational approaches matched to reference databases (Uppal et al., 2017). Additionally, metabolic pathway analysis is dependent upon complete and correct knowledge of metabolic pathways. Many known and unknown metabolites have not yet been mapped to referent metabolic pathways. Another major limitation is our reliance on multivariate techniques that do not allow for traditional covariate adjustment (e.g., age and sex differences in gray matter). The multivariate techniques such as PLS-DA and xMWAS are very good when the number of predictor variables outweigh the sample size (p > n) and when the predictors are highly correlated, the scenario we have here. However, these methods cannot adjust for confounders and this is a general limitation of the multiomics field. We did explore the possible impact of confounding by age and sex by examining their associations with the CSF metabolites in the cognitively normal controls. We found little to no overlap in metabolic pathways of those results and our primary results leading us to believe that confounding by age and sex may be underwhelming. Lastly, our approach was completely data-driven and relied on making multiple statistical comparisons, and thus our results should be replicated. Our sensitivity analysis suggested that our primary metabolic pathway findings were robust in our dataset, but independent validation is needed. Also, if critical brain regions were omitted, the analyses may not reveal the most appropriate metabolic pathways. Future research may consider specifying brain regions *a priori*. However, many of our results reflect similar metabolic pathways found in other studies, and thus provides additional support that the metabolite associations with brain regions are likely to be relevant to cognition. Despite these limitations, we feel our analysis found novel links to disrupted metabolic activity associated with specific areas of the human MCI brain, findings that would be difficult to reach using a non-multiomics approach.

In summary, a multiomics analysis of structural MRI brain imaging and CSF metabolomics revealed novel links between the urea cycle and amino acid metabolism and neurocognitive decline in MCI-susceptible areas of the brain. Similar approaches that integrate ‘big-data’ sources may offer novel insight into the pathogenic systems of MCI and other neurodegenerative diseases. The emerging field of imaging genomics – integrating brain imaging with high-resolution genome-wide data – is one approach that has helped narrow the study of candidate genes that may affect and predict early AD (Thompson et al., 2010; Zhang et al., 2014). Paired with other large data methods such as imaging, genetics, or the microbiome, metabolomics may continue to play an integral role in the future understanding, treatment, and prevention of cognitive decline.

## Data Availability Statement

The raw data supporting the conclusions of this article will be made available by request through the SAGE AD portal at: https://adknowledgeportal.synapse.org/Explore/Studies/DetailsPage?Study=syn18909507.

## Ethics Statement

The studies involving human participants were reviewed and approved by the Emory University Institutional Review Board. The patients/participants provided their written informed consent to participate in this study.

## Author Contributions

RE, KU, and IH: conceptualization. RE, KU, MS, and IH: methodology and formal analysis. IH: funding acquisition. RE, MS, and IH: project administration and data curation. KU, DJ, and IH: resources. RE and KU: software. KU, ZQ, DJ, and IH: supervision. RE: writing original draft. All authors: writing review and editing.

## Conflict of Interest

The authors declare that the research was conducted in the absence of any commercial or financial relationships that could be construed as a potential conflict of interest.

## Publisher’s Note

All claims expressed in this article are solely those of the authors and do not necessarily represent those of their affiliated organizations, or those of the publisher, the editors and the reviewers. Any product that may be evaluated in this article, or claim that may be made by its manufacturer, is not guaranteed or endorsed by the publisher.
