# Supplementary material for: Multiomics Analysis of Structural Magnetic Resonance Imaging of the Brain and Cerebrospinal Fluid Metabolomics in Cognitively Normal and Impaired Adults
Source: Front Aging Neurosci. 2022 Jan 25;13:796067. doi: 10.3389/fnagi.2021.796067 (PMC8822333; doi:10.3389/fnagi.2021.796067)
Supplement: Supplementary file 1 [file Data_Sheet_1.PDF]

EMORY UNIVERSITY SCHOOL OF MEDICINE<br>Department of Medicine	**Clinical Biomarkers Laboratory**<br>**Division of Pulmonary Allergy and Critical Care Medicine**<br>**615 Michael St. Ste. 225, Atlanta GA, 30322**

Title: Quality control and data analysis standard operating procedure for high-resolution metabolomics

**SOP:** HRM_DataAnalysis_092017_v2<br>Revision: 1	**Date effective:** September 2017

Summary

An adaptive processing software package, apLCMS (http://web1.sph.emory.edu/apLCMS/), designed for use with high-resolution mass spectrometry data, is used for noise removal and for feature extraction, alignment, and quantification (Yu 2009). Each metabolic feature is defined by a unique combination of *m/z* and retention time (RT). To enhance the feature detection process and perform quality evaluation, systematic data re-extraction and statistical filtering is performed using xMSanalyzer (Uppal 2013; http://sourceforge.net/projects/xmsanalyzer/). Each sample is analyzed in triplicate on the instrument, and coefficient of variation (CV) is used to evaluate the quality of all *m/z* features. Pearson correlation within technical replicates is used to evaluate the quality of samples. The measurements from the technical replicates are median summarized. Batch-effect correction is performed using ComBat (Johnson 2007). The features are annotated using the R package xMSannotator, which employs a multi-level clustering procedure based on intensity across all samples, retention time, mass defect, and isotope/adduct patterns (Uppal 2017). Additionally, xMSannotator uses metabolic pathway associations to assign confidence levels for database matches. Confidence levels range from zero to three, designating annotations from no confidence to high confidence, which reduces the risk of false annotations and allows prioritization of computationally derived annotations for further experimental evaluation and confirmation (Uppal 2017).

A. File conversion (.raw -> .CDF)

1. Once a batch is done, copy the raw files to the network Archive
   a. Be sure to create a folder under your name and another folder with the study name
      i. G:\Medicine\Pulmonary_ISILON\Research\Jones_Lab_Orbitrap\raw\[your name]\[study name]
      ii. This will ensure a copy of the raw files are kept in an archive database in case an error occurs later in the analysis
2. Raw files located on the hard drive are then converted to cdf files through Xcalibur
   a. Click on View > Roadmap view > Tools > File Converter

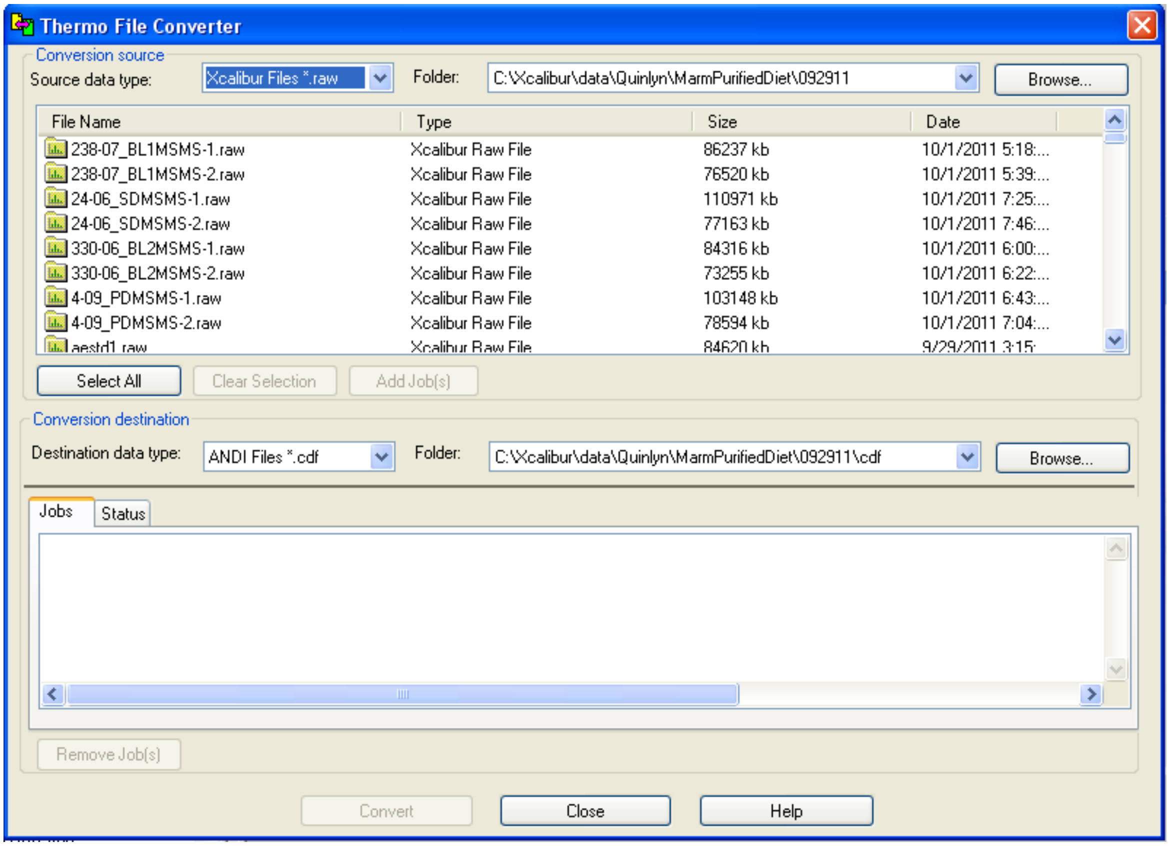

Figure 1. Screenshot of Thermo File Converter

3. Browse for the location of the RAW files
    a. RAW files found on the hard drive → converting files are quicker if left on the computer instead of transferring over the network
        i. C:\\Xcalibur\data\[choose correct folder]
4. Browse for the location of where the cdf files will be placed after conversion
    a. C:\\Xcalibur\data\cdf\[choose or create correct folder]
5. In "Thermo File Converter" → Hit "Select All" then hit "Add jobs"
6. At the bottom of the screen, click on the "convert" button
7. After all files are converted, copy .cdf files to the network
    a. G:\Pulmonary\Research\Jones Lab\Orbitrap\cdf\[choose folder]
    b. Delete both the raw and cdf files on the Orbitrap computer to make room for more data

B. Daily quality control

Each sample is analyzed in triplicates on the instrument. A quality control procedure based on XCMS and a set of confirmed metabolites and internal standards is used to evaluate the data quality of each batch with respect to: number of features detected, missing values, mass accuracy (threshold: <5 ppm), Pearson correlation within technical replicates (threshold: 0.9), and average coefficient of variation of feature intensities within replicates (threshold: <30%). Samples are re-analyzed on the instrument if the data does not meet the defined criteria.

Batch summary	batch1	batch2	batch3	batch4	batch5	batch6
Date	2017-10-16	2017-10-16	2017-10-16	2017-10-17	2017-10-19	2017-10-20
Time	1:46:10 PM	2:04:24 PM	2:23:01 PM	11:20:28 AM	11:52:39 AM	10:03:23 AM
Total number of features	1664	1878	2000	1907	2173	2098
4. Number of non-zero features	1615	1121	1968	1209	2149	2046
Number of features present in at least 90% of the samples	1661	1876	1998	1905	2173	2095
Number of features present in at least 50% of the samples	1664	1878	2000	1907	2173	2098
Number of target features detected	8	9	9	9	9	9
Average retention time difference in target features (< 30 s)	5.883	6.29	5.742	5.479	5.393	5.788
Average mass error in target features (< 5 ppm)	1.39	1.561	1.413	1.252	0.88	0.7798
Mean median technical replicate CV (median CV of each feature < 0.3)	0.1245	0.1551	0.1584	0.1474	0.1566	0.1466
Mean mean technical replicate Pearson correlation coefficient (> 0.9)	0.9859	0.9499	0.9765	0.9605	0.9781	0.9858
Batch mean median intensity	3239526.85	3538214.35	3421622.52	3226287.59	3612469.21	3301900.04

Figure 2. Sample output report from the quality control R script. Green means the values in individual batches meet the defined criteria.

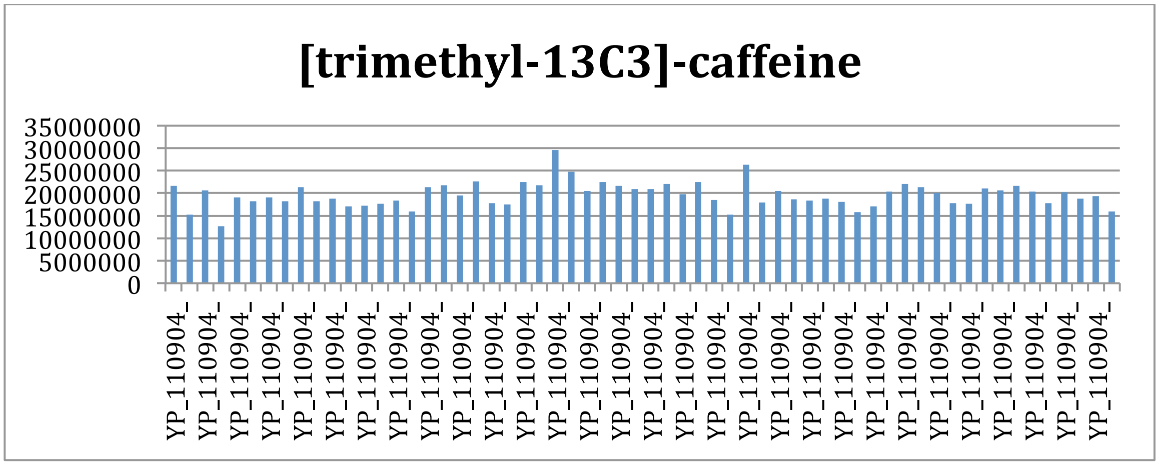

Figure 3. Example of the caffeine standard, m/z 198.0965, for the samples in the batch

1. Total Ion intensity is also a good indicator on whether the batch was interrupted midway or not.
   a. Scroll to the bottom
   b. In the empty box below Column E, type the following: =sum(E2:E[insert whatever the last row # is]);   Example: =sum(E2:E8584)
2. Do this for all the runs and graph Total sum vs file names

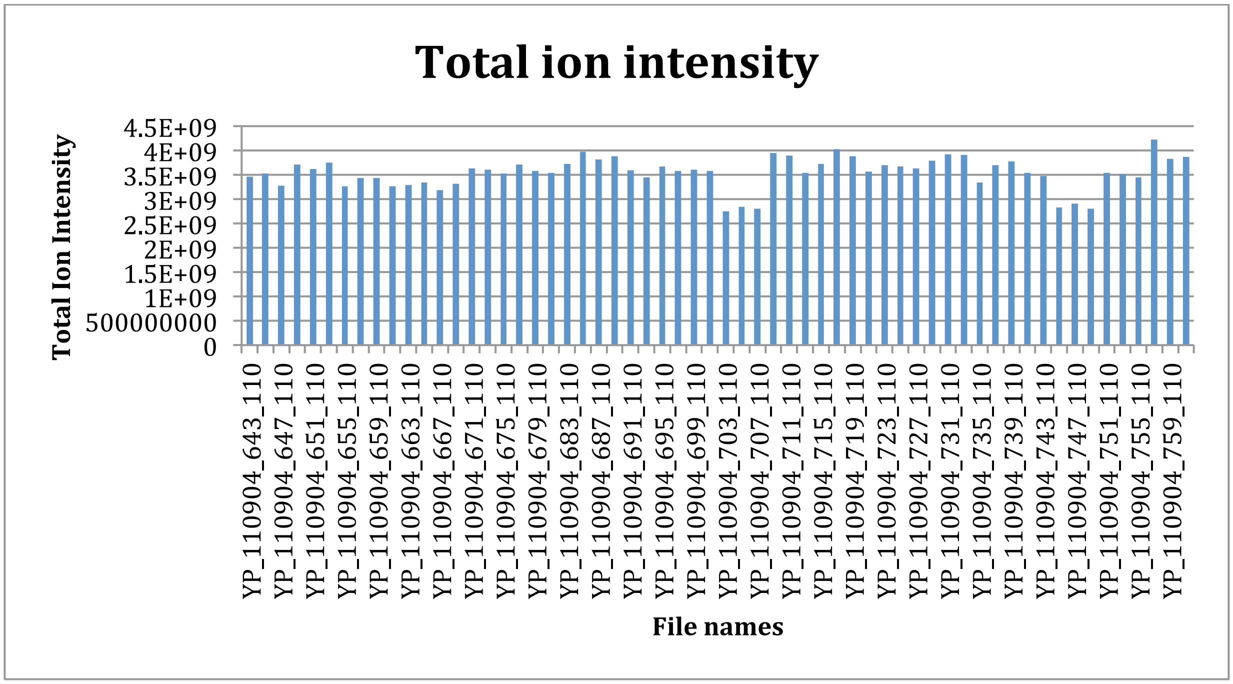

Figure 4. Total ion intensity for one batch

C. Data extraction using apLCMS with xMSanalyzer

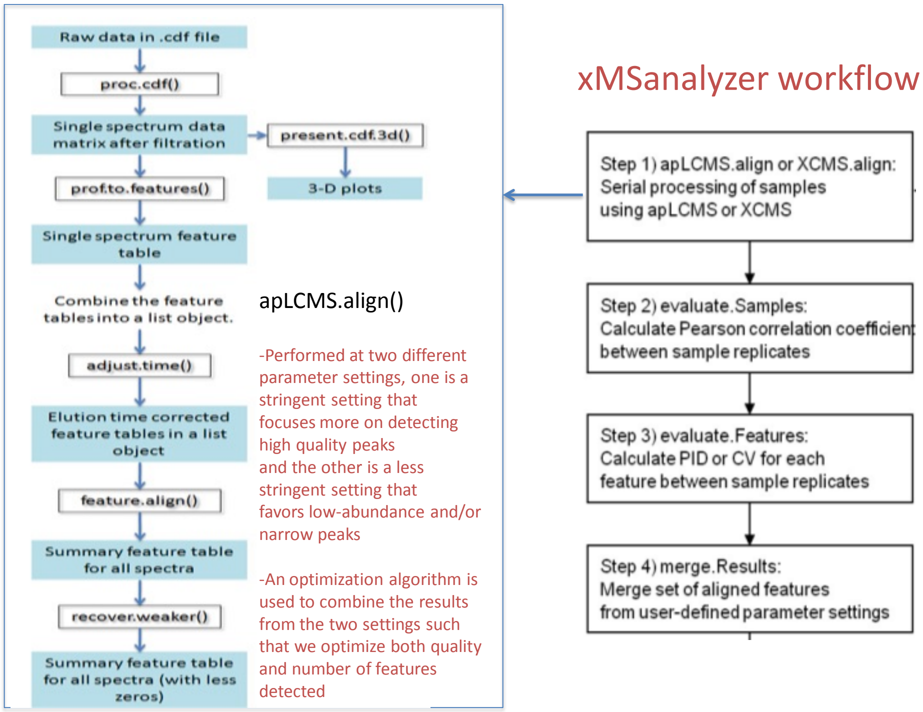

Figure 5. Overview of data extraction: steps involved in apLCMS and xMSanalyzer

1. On the Data processing computer (located outside in the lab area) → Double click on the “R” icon
    a. copy the folder with the .cdf from the network onto the data processing computer’s desktop
    b. apLCMS analyzes the data a quicker speed if file is located on the hard drive
2. apLCMS (Yu 2009) is an R package that performs peak detection, noise removal, peak quantification, peak alignment, and recovery of weak signals. The output of apLCMS includes retention times, m/z features that appeared in the LCMS run, and also the ion intensities of each of these m/z features for every sample.

3. File > Open Script > Desktop > R script for apLCMS (Figure 6). The script will perform peak detection and generate feature tables using multiple parameter settings.
4. Once the necessary changes are made, highlight all and right click and click on “run line”
5. The output of files will be located in the directory URL that was provided in the script
6. The feature tables will be located in the apLCMSuntargeted*.txt file which can be opened in Excel

```
library(apLCMS)
numnodes<-4
###apLCMS code
cdfloc<-"E:\\Hajjar_plasma\\mzXML\\hilicpos\\"
apLCMSoutloc<-"E:\\Hajjar_plasma\\mzXML\\hilicpos\\apLCMSv6.3.3_untargeted\\"
dir.create(apLCMSoutloc)
file.pattern = ".mzXML"
cdf.files<-list.files(cdfloc,file.pattern)
min.pres<-c(0.5,0.8)
min.run<- c(4,3)
min.exp<-round(0.1*length(cdf.files),0)
mz.tol<-2.5e-6
align.mz.tol<-10e-6
align.chr.tol = 30
match_tol_ppm=5
baseline.correct.noise.percentile = 0.25
shape.model = "bi-Gaussian"
baseline.correct = NA
peak.estim.method = "moment"
min.bw = NA
max.bw = NA
sd.cut = c(0.125, 15)
sigma.ratio.lim = c(0.33,3)
subs = NA
max.align.mz.diff = 0.01
pre.process = FALSE
recover.mz.range = 10e-6
recover.chr.range = 45
use.observed.range = FALSE
recover.min.count = 1
new_feature_min_count=4
component.eliminate= 0.01
moment.power=2
for(ii in 1:length(min.pres)){
#call cdf.to.ftr for extraction
print(paste("Settings", ii, "of", length(min.pres), sep=" "))
aligned.hyb<-cdf.to.ftr(folder=cdfloc,min.exp=min.exp,min.pres = min.pres[ii], min.run = min.run[ii],
mz.tol = mz.tol, align.mz.tol = align.mz.tol, align.chr.tol = align.chr.tol, n.nodes=numnodes, file.pattern=file.pattern,
baseline.correct.noise.percentile=baseline.correct.noise.percentile,
shape.model = shape.model, baseline.correct = baseline.correct, peak.estim.method = peak.estim.method,
min.bw = min.bw, max.bw = max.bw, sd.cut = sd.cut, sigma.ratio.lim = sigma.ratio.lim, subs = subs,
max.align.mz.diff = max.align.mz.diff, pre.process = pre.process, recover.mz.range = recover.mz.range,
recover.chr.range = recover.chr.range, use.observed.range = use.observed.range, recover.min.count =
recover.min.count, component.eliminate=0.01, moment.power=2)
finalfeattable<-aligned.hyb$final.ftrs
setwd(apLCMSoutloc)
fname1<-
paste("apLCMSuntargetedv6.3.3_min.run",min.run[ii],"min.pres",min.pres[ii],"min.exp",min.exp,"mztol",mz.tol,"alignm
ztol",align.mz.tol,"alignchr",align.chr.tol,".txt",sep="")
write.table(finalfeattable,file=fname1,sep="\t",row.names=FALSE)
}
```

Figure 6. R script file for apLCMSv6.3.3 program

7. Once the feature tables have been generated at multiple parameter settings using apLCMS, xMSanalyzer is used for:
    a. Optimizing peak detection by merging results from different parameter settings
    b. Quality evaluation of samples and features
        - Stage 1 includes feature quality evaluation (based on coefficient of variation within technical replicates) and sample quality evaluation (based on Pearson correlation within technical replicates)
        - The software will discard any feature with an average median coefficient of variation within technical replicates greater than 75%. The threshold is user-defined.
        - The software will discard any samples with an average Pearson correlation within technical replicates less than 0.7
        - Stage 4a includes a PDF file with a series of histograms, barplots, and dot plots to evaluate data quality
    c. *m/z* calibration using internal standards and confirmed metabolites
    d. Measurements from technical replicates are summarized such that at least 2 out of 3 replicates have a non-missing value
    e. Batch-effect evaluation and correction (Stage 4b)
        - Batch-effect evaluation is performed using PCA.
        - Batch-effect correction is performed using ComBat (Johnson 2007, Biostatistics) implemented in the "sva" package in R Bioconductor.
        - PCA plots are again generated to verify correction of batch-effects

8. Annotation: xMSannotator (Uppal 2017; Analytical Chemistry) R package is used for computational annotation of features. The algorithm uses a multi-level clustering procedure based on intensity across all samples, retention time, mass defect, and isotope/adduct patterns (Uppal 2017). Additionally, xMSannotator uses metabolic pathway associations to assign confidence levels for database matches. Confidence levels range from zero to three, designating annotations from no confidence to high confidence, which reduces the risk of false annotations and allows prioritization of computationally derived annotations for

further experimental evaluation and confirmation (Uppal 2017). Sample script for xMSannotator is shown in Figure 8.

```
library(apLCMS)
library(RColorBrewer)
library(xMSanalyzer)
library(xMSannotator)
data(keggCompMZ)


source("K:\\Medicine\\Pulmonary_ISILON\\Research\\Jones_Lab\\Orbitrap\\Rscripts_data_extraction\\Windows\\xMSanalyzer_2.0.7.999_debug41.R")
data(example_target_list_pos)
data(example_target_list_neg)
###Input parameters###########
#1) cdfloc: The folder where all CDF files to be processed are located. For example "C:/CDF/"
# Note: set cdfloc=NA if the cdf files are already aligned using apLCMS and the results  exist in apLCMS.outloc

cdfloc=NA #"E:\\24_25_18\\c18neg\\"

#Note: Feature table at each individual parameter setting (just like apLCMS)
#2) apLCMS.outloc: The folder where alignment output will be written. For example "C:/CDFoutput/"
apLCMSoutloc="E:\\Hajjar_plasma\\mzXML\\c18neg\\apLCMSv6.3.3_untargeted\\"

#3) xMSanalyzer.outloc: The folder where xMSanalyzer output will be written. For example "C:/xMSanalyzeroutput/"
xMSanalyzeroutloc="E:\\Hajjar_plasma\\mzXML\\c18neg\\xMSanalyzerv2.0.8debug41\\"

#4) Sequence file path; Need for batch-effect evaluation; eg: "C:/Documents/Emory/JonesLab/Projects/pos/sequence_file_pos.txt"
#Column A: Names matching .cdf or .mzXML files
#Column B: Sample ID/name
#Column C: Batch (column should be labeled "Batch")
sample_info_file<-"E:\\Hajjar_plasma\\mzXML\\Hajjar_plasma_mapping_c18neg.txt"

#5) reference chemicals; use NA for the example_target_list provided with the package
# eg:"C:/Documents/Emory/JonesLab/Projects/xMSanalyzer/valid_chem_mz.txt"
reference_chemicals_file<-NA

#6) Ionization mode: use "pos" for positive and AE; use "neg" for negative and C18
charge_type="neg"

#7) Length of chromatography: 300 for 5 min method; 600 for 10 min method
```

```
max_retention_time<-300

#8) file pattern: ".cdf" or ".mzXML"
filepattern=".mzXML"

#############################################END of Input
parameters##################################################################
dir.create(apLCMSoutloc,showWarnings=FALSE)
dir.create(xMSanalyzeroutloc,showWarnings=FALSE)
numnodes <- detectCores() - 1
numnodes<-round(numnodes*0.6)
########xMSanalyzer usage##################
if(max_retention_time>300){
alignchrtol = 45
}else{
alignchrtol = 30
}
        result<-try(
        {
        par(mfrow=c(2,2))
        pdf("Rplots.pdf")
          res.list<-xMSwrapper.apLCMS(cdfloc=cdfloc, apLCMS.outloc=apLCMSoutloc,
xMSanalyzer.outloc=xMSanalyzeroutloc, minexp.pct = 0.1, max.mz.diff = 15, max.rt.diff =
0.5*(max_retention_time), merge.eval.pvalue = 0.05, mergecorthresh = 0.7, deltamzminmax.tol = 100,
          num_replicates = 3,
          mz.tolerance.dbmatch = 15, adduct.list = c("M+H"), samp.filt.thresh = 0.7,  feat.filt.thresh = 75,
cormethod = "pearson", mult.test.cor = FALSE,
          missingvalue = 0, ignore.missing = TRUE, filepattern = filepattern,
        sample_info_file=sample_info_file,refMZ=reference_chemicals_file,refMZ.mz.diff=10,refMZ.time.diff=
NA,void.vol.timethresh=30,
        replacezeroswithNA=TRUE,charge_type=charge_type,plotEICs="target",rawprofileloc=cdfloc,peak.sc
ore.thresh=NA,reference_sample_index = NA, merge.pairwise = FALSE,
summarize.replicates=TRUE,summary.method="median",max.number.of.replicates.with.missingvalue=1,su
mmary.na.replacement="zeros",db_name=c("KEGG","HMDB","LipidMaps"),qc_label="q3june2014",data.nor
m.pipeline="AC"
          #end
          )
try(dev.off(),silent=TRUE)
        })
```

Figure 7. R script file for xMSanalyzerv2.0.7 program

```
library(xMSannotator)
library(xmsPANDA)

#Package data files
data(example_data)  #example peak intensity matrix
data(adduct_table)
data(adduct_weights)
data(customIDs)  #example for custom IDs
data(customDB)   #example for custom DB
#data(hmdbAllinf)
#data(keggotherinf)
#data(t3dbotherinf)


###########Parameters to change##############
#dataA<-example_data #use example data provided with the package
#OR
dataA<-
read.table("E:\\Hajjar_plasma\\mzXML\\c18neg\\xMSanalyzerv2.0.8debug41\\Stage4b\\ComBat_mzcalibrat
ed_untargeted_mediansummarized_featuretable.txt",sep="\t",header=TRUE)

#output location
outloc<-"E:\\Hajjar_plasma\\mzXML\\c18neg\\xMSannotatortestv1.3.1\\"

max.mz.diff<-10  #mass search tolerance for DB matching in ppm
max.rt.diff<-10 #retention time tolerance between adducts/isotopes
corthresh<-0.7 #correlation threshold between adducts/isotopes
max_isp=5 #maximum number of isotopes to search for
mass_defect_window=0.01 #mass defect window for isotope search

num_nodes<-2   #number of cores to be used; 2 is recommended for desktop computers due to high
memory consumption

db_name="HMDB" #other options: HMDB,Custom,KEGG, LipidMaps, T3DB
status=NA #other options: "Detected", NA, "Detected and Quantified", "Expected and Not Quantified"
num_sets<-300 #number of sets into which the total number of database entries should be split into;

ionization_mode<-"neg" #pos or neg, options for ionization mode

#provide list of database IDs (depending upon selected database) for annotating only specific metabolites
customIDs<-NA #c("HMDB15352","HMDB60549","HMDB00159","HMDB00222");
```

```
read.csv("/Users/mzmatch_95stdmx_HMDBIDs.csv")

#provide your own custom database to be used for annotation
#set db_name="Custom" if you use this option
#Format: ID, Name, Formula, MonoisotopicMass
customDB<-NA
#read.table("/Users/karanuppal/Documents/Emory/JonesLab/Projects/xMSannotator/IROA/IROA_customD
B_xMSannotator_plate1.txt",sep="\t",header=TRUE) #custom database; default NA

#number of technical replicates
num_replicates<-1

##########################
if(ionization_mode=="neg"){
filter.by=c("M-H")
queryadductlist=c("M-H","M-H2O-H","M+Na-2H","M+Cl","M+FA-H")
}else{
filter.by=c("M+H")
queryadductlist=c("M+2H","M+H+NH4","M+ACN+2H","M+2ACN+2H","M+H","M+NH4","M+Na","M+ACN+H"
,"M+ACN+Na","M+2ACN+H","2M+H","2M+Na","2M+ACN+H","M+2Na-H","M+H-H2O","M+H-2H2O")
}
dataA<-unique(dataA)
print(dim(dataA))
print(format(Sys.time(), "%a %b %d %X %Y"))

system.time(annotres<-
multilevelannotation(dataA=dataA,max.mz.diff=max.mz.diff,max.rt.diff=max.rt.diff,cormethod="pearson",nu
m_nodes=num_nodes,queryadductlist=queryadductlist,
mode=ionization_mode,outloc=outloc,db_name=db_name,
adduct_weights=adduct_weights,num_sets=num_sets,allsteps=TRUE,
corthresh=corthresh,NOPS_check=TRUE,customIDs=customIDs,missing.value=NA,deepsplit=2,networktyp
e="unsigned",
minclustsize=10,module.merge.dissimilarity=0.2,filter.by=filter.by,biofluid.location=NA,origin=NA,status=stat
us,boostIDs=NA,max_isp=max_isp,
customDB=customDB,
HMDBselect="union",mass_defect_window=mass_defect_window,pathwaycheckmode="pm",mass_defect_
mode="pos")
)
print(format(Sys.time(), "%a %b %d %X %Y"))
```

Figure 8. R script file for xMSannotatorv1.3.1 program

Recommendations for additional data processing:

A $\log_2$ transformation can be applied to reduce heteroscedasticity and normalize results. Quantile normalization reduces between-sample variability (Patel 2015). To increase confidence for selection of discriminating metabolites, data can be filtered based on missing values criteria and only those features present in at least 80% of either cases or controls and present in at least 50% of all samples can be included in downstream statistical analyses. Univariate and multivariate statistical methods such as LIMMA ($p<0.05$; FDR<0.1) and partial least squares-discriminant analysis (PLS-DA; variable importance for projection >2) can be used for feature selection. Two-way hierarchical clustering analysis and principal component analysis can be used for visualizing the clustering patterns of samples and features. Additional graphical plots such as Manhattan plots (e.g. $-\log_{10}p$-value vs *m/z*), volcano plots ($-\log_{10}p$-value vs fold-change), boxplots, and barplots can be used for visualizing the differential expression pattern of selected features. Mummichog can be used for pathway enrichment analysis.

References:

Johnson WE, Li C, Rabinovic A. Adjusting batch effects in microarray expression data using empirical Bayes methods. *Biostat Oxf Engl*. 2007;8(1):118-127. doi:10.1093/biostatistics/kxj037
Li S, Park Y, Duraisingham S, et al. Predicting network activity from high throughput metabolomics. *PLoS Comput Biol*. 2013;9(7):e1003123. doi:10.1371/journal.pcbi.1003123

Patel RM, Roback JD, Uppal K, Yu T, Jones DP, Josephson CD. Metabolomics profile comparisons of irradiated and nonirradiated stored donor red blood cells. *Transfusion (Paris)*. 2015;55(3):544-552. doi:10.1111/trf.12884

Uppal K, Walker DI, Jones DP. xMSannotator: An R Package for Network-Based Annotation of High-Resolution Metabolomics Data. *Anal Chem*. 2017;89(2):1063-1067. doi:10.1021/acs.analchem.6b01214
Uppal K, Soltow QA, Strobel FH, et al. xMSanalyzer: automated pipeline for improved feature detection and downstream analysis of large-scale, non-targeted metabolomics data. *BMC Bioinformatics*. 2013;14:15. doi:10.1186/1471-2105-14-15

Yu T, Park Y, Johnson JM, Jones DP. apLCMS--adaptive processing of high-resolution LC/MS data. *Bioinforma Oxf Engl*. 2009;25(15):1930-1936. doi:10.1093/bioinformatics/btp291

SOP Details and Version Information

**Created by:** Karan Uppal	**Date:** 29 September 2017
