# Supplementary material for: Multiomics Analysis of Structural Magnetic Resonance Imaging of the Brain and Cerebrospinal Fluid Metabolomics in Cognitively Normal and Impaired Adults
Source: Front Aging Neurosci. 2022 Jan 25;13:796067. doi: 10.3389/fnagi.2021.796067 (PMC8822333; doi:10.3389/fnagi.2021.796067)
Supplement: Supplementary file 2 [file Table_1.docx]

**Supplemental Methods and Findings**

**Supplemental Figure S1.** Metabolite intensity density plot before and after log_2_-transformation and quantile normalization:


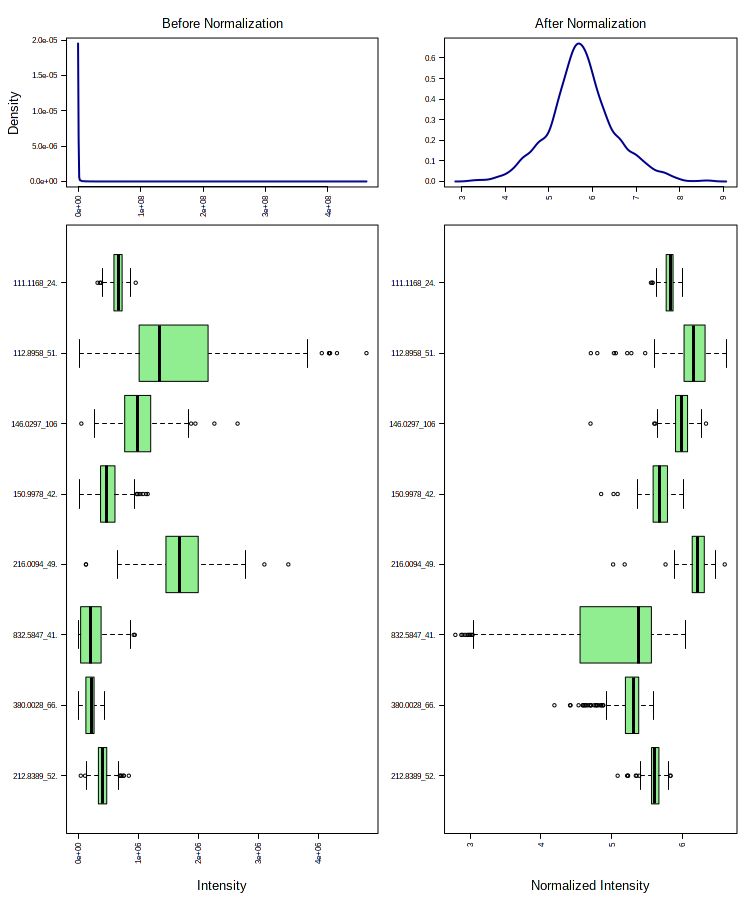


Per sample intensity density plot before and after log_2_-transformation and quantile normalization:


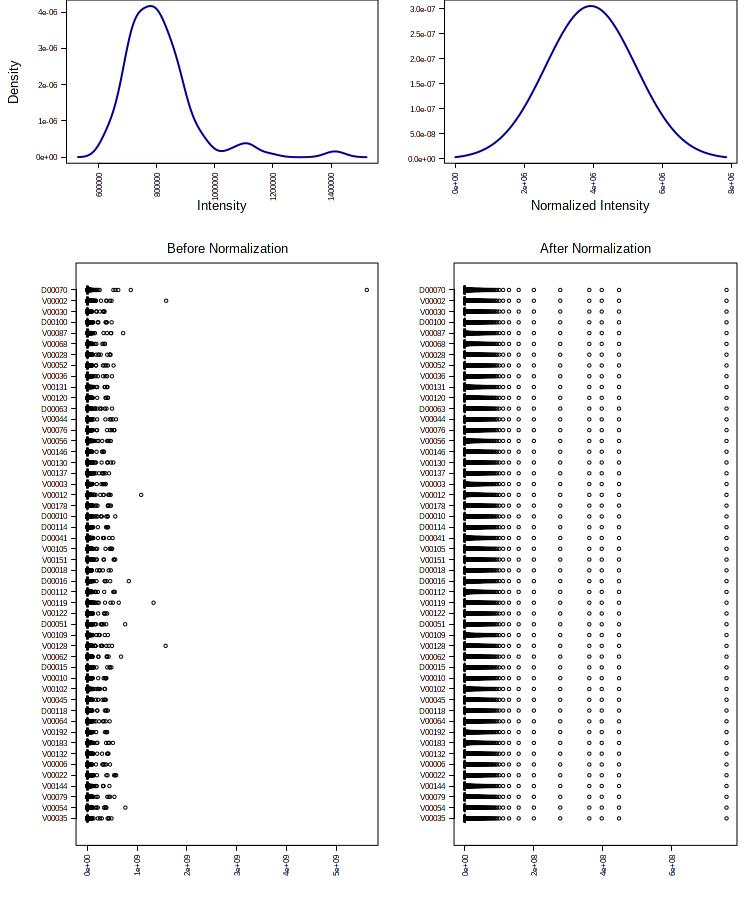


**Supplemental Figure S2**. Partial least squares discriminant analysis score plots for the top three PLS components from the Step 1 model – selecting MRI imaging voxels that discriminate between MCI and cognitively normal controls. Orange triangles are MCI cases and blue circles are cognitively normal controls.


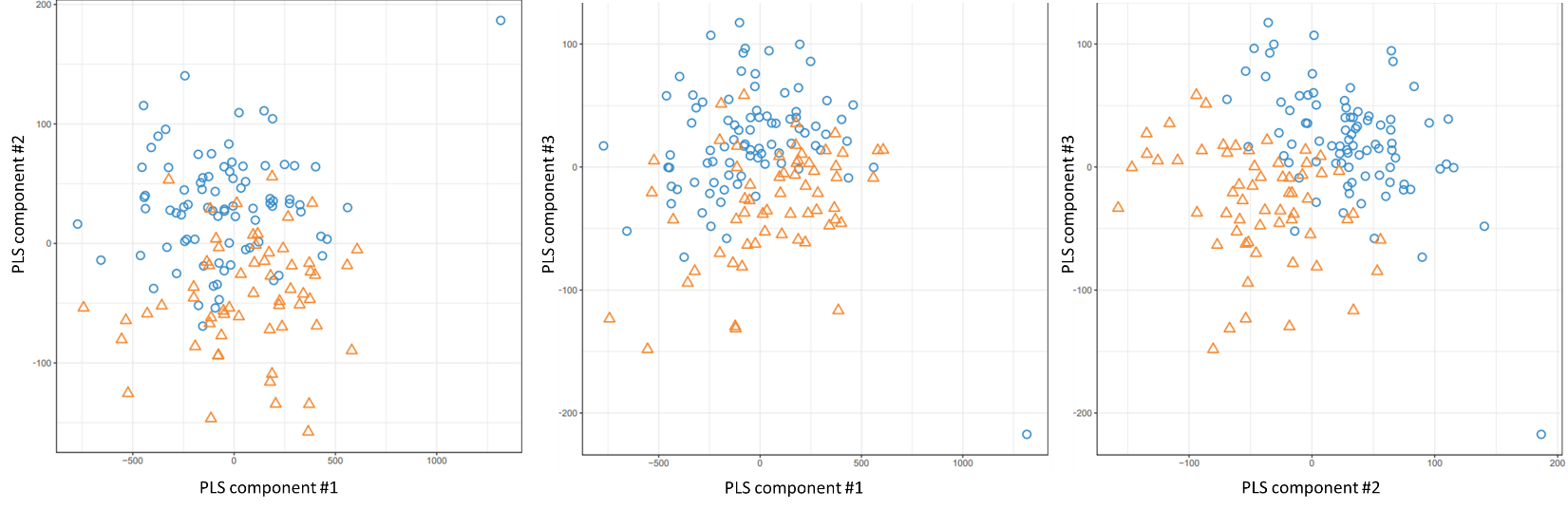


**Confounding Assessment by Age and Sex.**Since the multivariate methods we employed do not allow covariate adjustment, we assessed the possible impact of confounding of our primary metabolic pathway results by comparing the metabolites and metabolic pathways associated with age or sex among the cognitively normal population to our primary findings. Less overlap would suggest less confounding and greater overlap would suggest greater confounding. Using the 9,804 CSF metabolites, we chose the top 463 metabolites with the lowest P-value associated with continuous age via a linear regression and the top 463 metabolites with the highest VIP associated with sex via a PLS-DA. We chose to limit our analyses to 463 in each group to make a fair comparison with our primary results which found 463 metabolites linked to MRI imaging voxels in Step 2. Comparing the 463 metabolites in our primary results to the 463 significant with age, only 54 (11.7%) overlapped. Even fewer, only 45 (9.7%), overlapped with sex. Zero of the metabolites in Table 3 were associated with age or sex. Metabolic pathway analysis of the 463 metabolites associated with age showed no overlap in metabolic pathways (Supplemental Table 1) compared to our primary results – which found significant pathway enrichment in the Urea Cycle and a host of amino acids (Figure 1 and Table 3). In the pathways associated with sex, histidine metabolism and methionine and cysteine metabolism did overlap but were weaker and less consistent pathways found in our primary results (Supplemental Table 2). Based on these results, we concluded that confounding by age and sex was minimal and would not change our primary results or our conclusions.

**Supplemental Table 1.** The enriched metabolic pathways from the top 463 associated CSF metabolites with continuous Age using in a linear regression model among the cognitively normal controls.
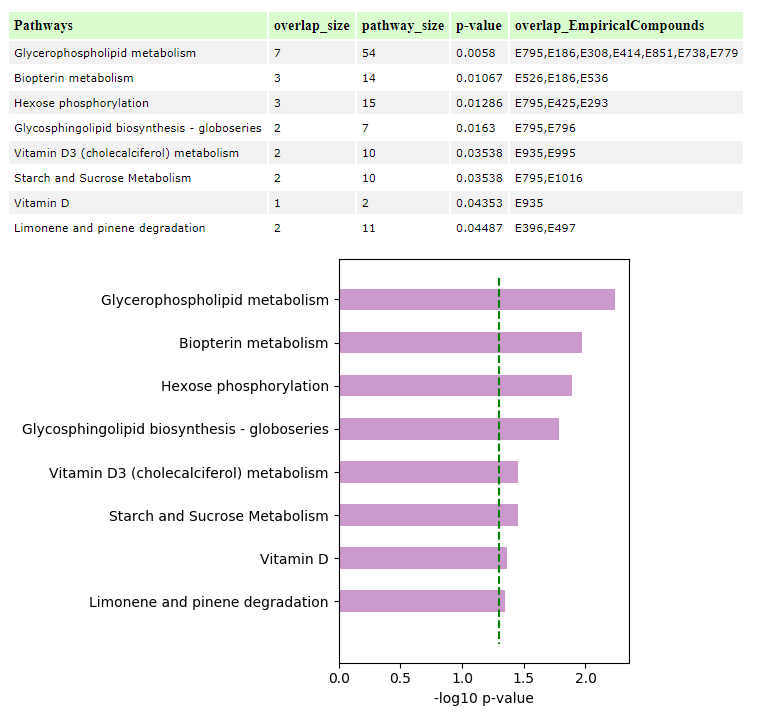


**Supplemental Table 2**. The enriched metabolic pathways from the top 463 associated CSF metabolites with Sex using in a PLS-DA regression among the cognitively normal controls.
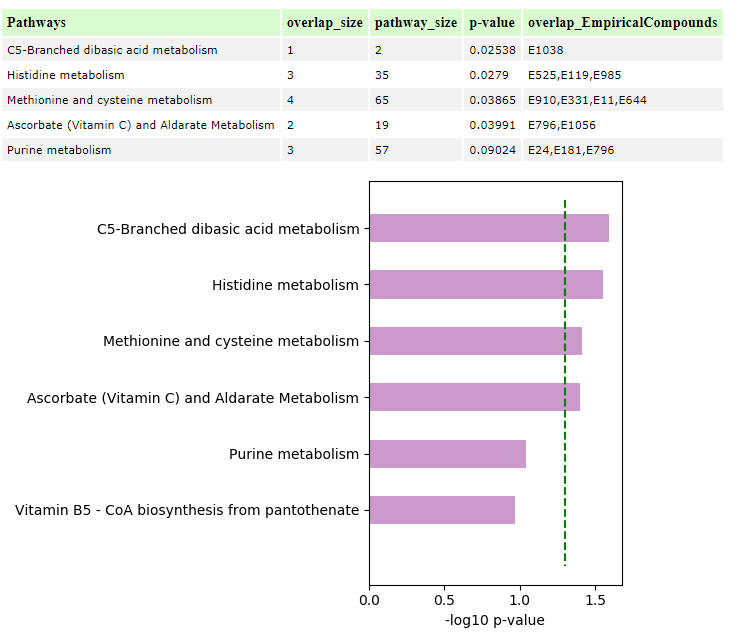


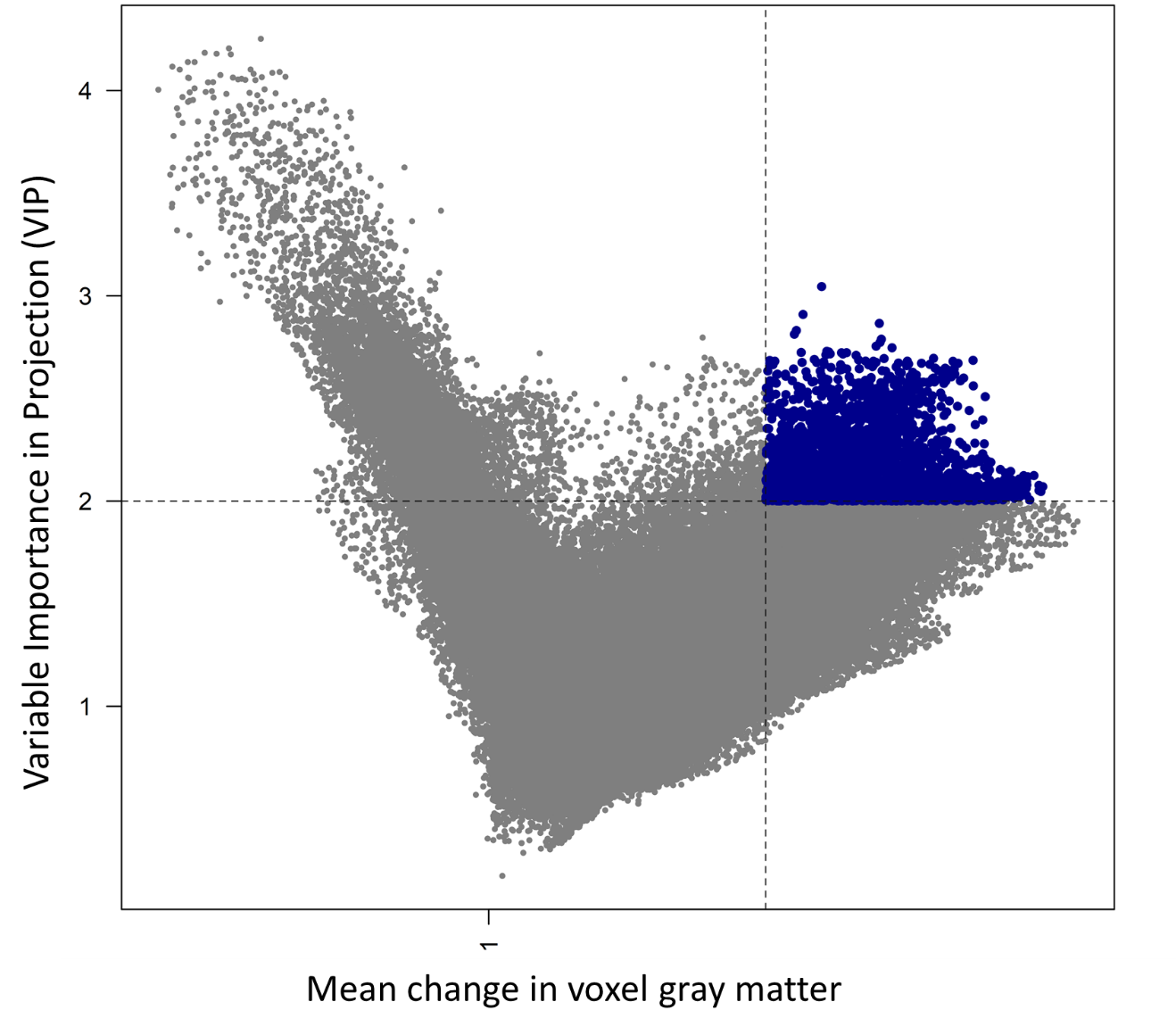


**Supplemental Figure S3.** A volcano plot of the mean change in gray matter volume (MCI vs cognitively normal) on the x-axis and the variable importance in projection (VIP) from the partial least squares discriminant analysis on the y-axis. The blue highlighted points depict the 2,375 voxels out of 247,941 that met our predetermined threshold of VIP>2 and mean change >2% in the direction of greater gray matter in cognitively normal subjects. A total of 15,629 voxels met the VIP>2 cutoff, but only 2,424 met that and the mean change threshold. Of those, 49 voxels showed higher levels of mean gray matter in MCI subjects and were excluded from further analyses.


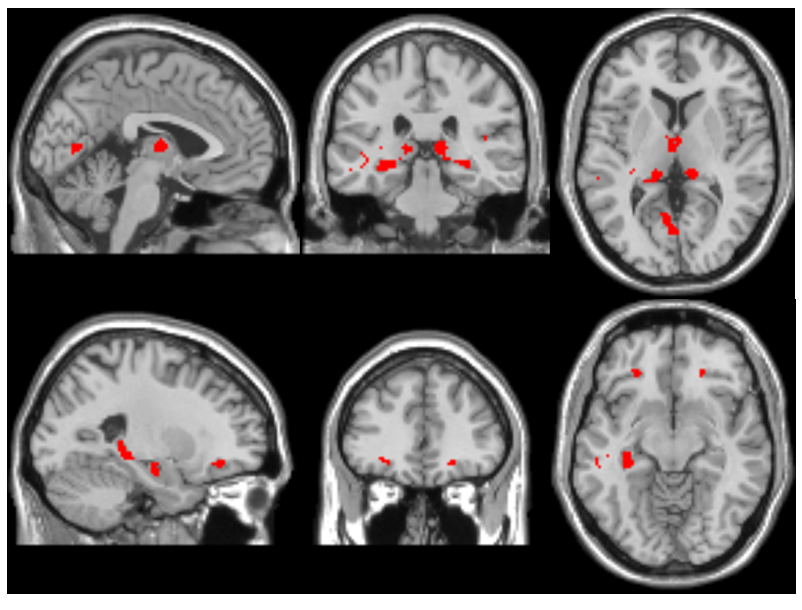


**Supplemental Figure S4**. The brain neuroimaging atlas of the 2,375 voxels (highlighted in red) that the partial-least squares discriminant analysis identified as having lower gray matter in MCI vs healthy subjects. All voxels met a cutoff criteria of VIP≥2 and >2% mean difference in gray matter volume.


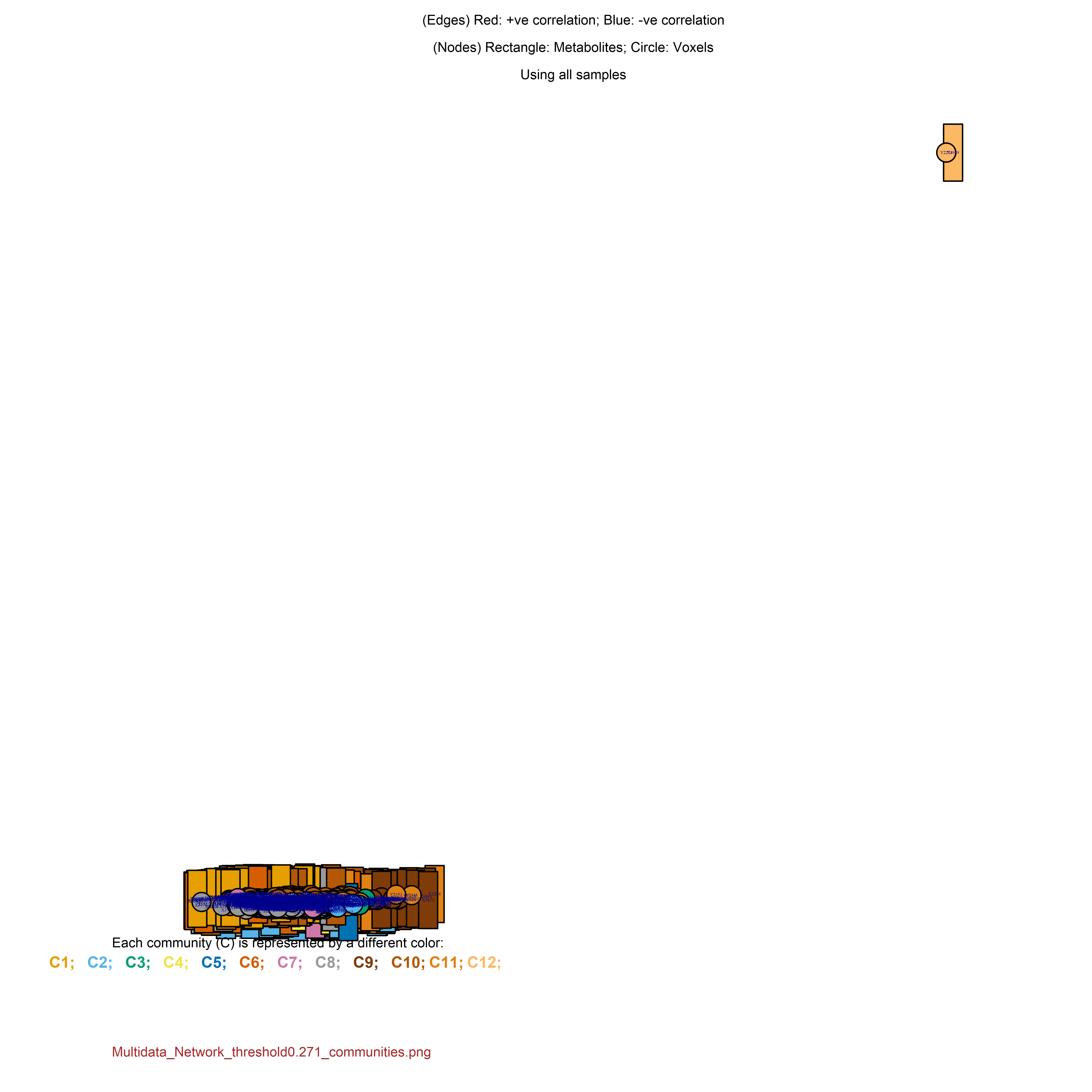


**Supplemental Figure S5.** The unaltered figure with 12 imaging-metabolite clusters xMWAS identified using an absolute value Pearson r cutoff of 0.271. The circles (n=2,375) are MRI brain imaging voxels and the rectangles (n=463) are cerebrospinal fluid metabolites. As is apparent, the vast number of connections between imaging voxels and CSF metabolites necessitated a modified figure with a stricter Pearson’s r value (r>0.35) to show the clusters (Figure 1A).

**Panel (a)**

**Panel (b)**

**Supplemental Figure S6a-b.** Scatter plots of voxel-metabolite correlations, as estimated by xMWAS, in the training set and the identical voxel-metabolite correlations in the validation set with a fitted linear regression and R^2^ value. **Panel (a)** includes all voxel-metabolite links that met the xMWAS parameters (r >│0.271│with a P value<0.05) in the training set. **Panel (b)** includes only the voxel links with 17 of the 20 metabolites found via the Step 3 metabolic pathway analysis that met the xMWAS parameters in the training set. Indoleacetic acid, 5-hydroxyindoleacetate, and S-adenosylhomocysteine did not meet the parameter thresholds in the training set and were thus excluded.
